# GPT-4 shows potential for identifying social anxiety from clinical interview data

**DOI:** 10.1038/s41598-024-82192-2

**Published:** 2024-12-16

**Authors:** Julia Ohse, Bakir Hadžić, Parvez Mohammed, Nicolina Peperkorn, Janosch Fox, Joshua Krutzki, Alexander Lyko, Fan Mingyu, Xiaohu Zheng, Matthias Rätsch, Youssef Shiban

**Affiliations:** 1 Clinical Psychology Department, PFH University of Applied Sciences, Göttingen, Germany; 2 ViSiR, Reutlingen University, Reutlingen, Germany; 3 Institute of Artificial Intelligence, Donghua University, Shanghai, China

**Keywords:** Social anxiety, Anxiety, Artificial intelligence, Natural language processing, Generative pre-trained transformers, GPT-4, Psychology, Computer science, Health care

## Abstract

While the potential of Artificial Intelligence (AI)—particularly Natural Language Processing (NLP) models—for detecting symptoms of depression from text has been vastly researched, only a few studies examine such potential for the detection of social anxiety symptoms. We investigated the ability of the large language model (LLM) GPT-4 to correctly infer social anxiety symptom strength from transcripts obtained from semi-structured interviews. *N* = 51 adult participants were recruited from a convenience sample of the German population. Participants filled in a self-report questionnaire on social anxiety symptoms (SPIN) prior to being interviewed on a secure online teleconference platform. Transcripts from these interviews were then evaluated by GPT-4. GPT-4 predictions were highly correlated (*r* = 0.79) with scores obtained on the social anxiety self-report measure. Following the cut-off conventions for this population, an F_1_ accuracy score of 0.84 could be obtained. Future research should examine whether these findings hold true in larger and more diverse datasets.

## Introduction

Social anxiety disorder (SAD) is one of the most common psychological disorders globally. Individuals affected by it experience anxiety symptoms in social situations, especially when they fear negative judgment^1^. The lifetime prevalence for SAD varies by country, ranging between 5.5% in high-income and 1.6% in low/lower-middle income countries^2^. SAD is associated with considerable role impairment, resulting in a mean number of 24.7 days out of work per year, and even greater impairment in relationships and social situations^2^. Furthermore, SAD is associated with an elevated risk of developing depression and a more malignant course of depression than that observed in individuals without SAD^3^.

Social anxiety disorder (SAD) can be treated: A meta-analysis^4^ revealed medium-to-large effect sizes for psychotherapy in patients from the general population and primary care settings. SAD patients in secondary and tertiary care settings, however, were found to be more prone to a chronic course of the disease, which is indicative of a need for early discovery and intervention^4^.

In contrast, epidemiological studies and surveys show that only 35% of people with social anxiety disorder (SAD) have received a diagnosis for this condition, which would be a first step towards receiving treatment^5–7^. In fact, the mean delay between disorder onset and first treatment contact was 16 years for SAD^8^. Since social anxiety is also associated with worse educational performance, the impact on the affected individuals’ later lives can be severe, e.g., in terms of limiting their choice of profession^9^. Moreover, SAD is correlated with both loneliness and isolation^10^. These outcomes can be explained by the disorder-typical avoidance of social situations, which also has a detrimental effect on help-seeking behaviour^11^.

Research highlights that shame and stigma are the strongest barriers to seeking treatment for SAD^12^. This is consistent with the core symptoms of SAD: fear and avoidance of social situations, along with the dread of eliciting negative judgments from others, through behavior or by showing anxiety symptoms^1^.

The diagnosis of social anxiety disorder (SAD), however, requires the disclosure of anxiety symptoms in a clinical interview, with self-report questionnaires and stress tests providing ancillary indications of the disorder^13,14^. This makes the diagnostic process potentially challenging for individuals affected by SAD and might contribute to the avoidance of help-seeking, causing low diagnostic rates in the affected population.

Another factor contributing to this are the relatively low detection rates by general practitioners when it comes to anxiety disorders, which are particularly low if no further diagnostic tools were used^15^. SAD, in particular, was reported to go undetected in 97.8% of cases presenting in clinics^16^, making it the least detected anxiety disorder within this study. This prompts an inquiry into new technologies for making screenings for SAD available to larger populations and enhancing diagnostics.

The notion of using different AI algorithms and approaches for medical diagnosis and screening has become increasingly popular in recent years. Deep learning is one particular technique that has produced breakthrough results in several important domains, including speech recognition, natural language processing (NLP), and image classification^17^. In the medical sector, these systems have the potential to improve treatment decisions, lower costs associated with human experts, shorten clinical workflows, increase operational effectiveness, and improve diagnostic accuracy^18^.

Theoretically, an NLP model should be able to assess data from structured clinical interviews due to: (a) its natural language understanding capacities^19^ and (b) the potential to evaluate linguistic markers, which might also go beyond the scope of a human interviewer^20^. Indeed, a study which extracted linguistic markers for depression and anxiety from a clinical interview showed distinctions and overlaps between both of these concepts^21^.

If new digital screening methods prove accurate, they could pave the way for alternative approaches to detecting SAD, such as online-based systems or interviews with automatic agents. Given that many people with SAD prefer online contact over in-person interactions^22^, these technologies could potentially reduce impediments to an initial diagnosis. A prerequisite for this, however, is that NLP tools are accurate in their identification of symptoms and their severity.

### Current research

Over the last four years, the body of research about AI-driven diagnostic and screening systems for mental health disorders has been growing rapidly^23^. AI models for screening purposes can be classified as machine learning (ML), deep learning (DL), and natural language processing (NLP) models^24^, with NLP models offering the potential of comprehending and generating human-like language. According to a narrative review by Zhang et al.^19^, such NLP-models are primarily used for depression detection (45% of the studies) with social media posts as the source material (81% of studies). The validity of using social media posts for early screening is limited by two factors: (a) the format of the source material does not follow clinical standards that ensure that all symptoms are taken into account; and (2) the ground truth for “mental illness” is disputable (e.g., "posting in a subReddit for depression" or "using negative words"). In fact, only 2% of the studies evaluated by Zhang et al.^19^ used interviews as a source. Our most recent study evaluated the performance of different NLP models in detecting depression from transcribed interview data and found high accuracy for the untrained GPT-4 model (F_1_ score 0.73), as well as for the few-shots fine-tuning GPT-3.5 model (F_1_ score 0.82)^25^.

Zhang et al.^19^ showed that only 2% of the eligible studies on NLP-based disorder detection investigate anxiety disorders. Even in 2024, the focus appears to be on depression detection, leaving a notably smaller field of research for anxiety disorder detection:

Zarate et al.^26^ successfully used an NLP model for predicting self-reported anxiety disorder diagnosis from tweets (79.4–81.1% accuracy). Stade et al.^21^ managed to extract distinctive language patterns for anxiety and depression from clinical interview data. Rutowski et al.^27^ used a deep language model to analyze spontaneous speech for depression, generalized anxiety disorder or their comorbidity, with an accuracy varying from 0.50 up to 0.90. Burkhardt et al.^28^ managed to achieve an accuracy of 0.46 for detecting generalized anxiety disorder in text-based therapy sessions, while Wright-Berryman et al.’s^29^ logistic regression (LR) model managed to detect generalized anxiety with an accuracy of 0.7 in *n* = 2,416 interviews.

With regards to social anxiety disorder (SAD), there are even fewer studies testing the capacity of NLP systems. However, brain information flow^30^, various attribute data^31,32^, and mobility data^33^ have been used successfully for inferencing social anxiety.

Salekin et al.^34^ used different multiple instance learning (MIL) and supervised learning algorithms to make a distinction between students high in social anxiety vs. students low in social anxiety by analyzing the content of a speech stressor task, attaining F_1_ accuracies of 68.3—90.1%. Byers et al.^35^ employed deep learning and machine learning techniques to detect social anxiety from *n* = 10 transcribed interviews in a population of student veterans with PTSD with an accuracy of up to 61.2%.

In aggregate, NLP has made significant advances in the screening for mental illnesses in recent years, particularly due to certain approaches, such as long short-term memory networks (LSTMs), recurrent neural networks (RNNs), and transformers. Neural network architectures, known as recurrent neural networks (RNNs), are mostly used to identify patterns in sequential data by preserving a hidden state which stores information about prior sequence elements^36^. LSTMs are a specialized variant of RNNs, which are designated to overcome problems recurrent in RNNs in terms of capturing long-term dependencies, achieving this by using gated memory cells that store and retrieve information over time^37^. Therefore, LSTMs perform better in tasks involving natural language processing (NLP), such as speech recognition, language modeling, and context understanding, in which the ability to capture long-range dependencies is required^38^. Lastly, transformers, with their ability to manage long-range dependencies and efficiently parallelize computations, constitute a significant advancement, greatly outperforming RNNs and LSTMs in various tasks. Transformers are more effective because of their self-attention mechanism, which enables them to evaluate each word’s relevance in a phrase simultaneously rather than sequentially^39^.

### Research gap

While Byers et al.^35^ and Salekin et al.^34^ demonstrated AI models’ potential to infer social anxiety symptoms from speech data, further research is essential to advance this field. Both studies were conducted using student samples, highlighting the need to extend these investigations to a more diverse general population. With large language models (LLMs) showing promise in addressing various mental health concerns^19,25^, it is crucial to explore their capabilities for detecting and measuring social anxiety symptoms specifically. Like many prior studies, we will use self-report measures as our ground truth. Our methodology will focus on an interview-based format with open-ended questions about social anxiety symptoms, encouraging participants to produce free speech. This approach allows for an analysis of linguistic differences between individuals with and without social anxiety disorder (SAD). Additionally, we aim to assess the accuracy of symptom severity evaluations by correlating model predictions with self-reported ground truth values. Given GPT-4’s demonstrated capabilities in zero-shot depression detection in our prior research^25^, we have selected this specific model for our current study on social anxiety detection.

### Research question

The research question of the present study is as follows: Can GPT-4 accurately infer social anxiety disorder (SAD) symptom severity from semi-structured interview data?

## Methods

### Study design and outcomes

To test GPT-4’s ability to correctly infer social anxiety symptom strength from transcribed interviews, we chose a cross-sectional design, aligning with our prior research^21^. Based on the theory that NLP models, such as GPT-4, have the capacity to understand the content of clinical interviews and might even be able to detect further linguistic markers of mental disorders, we prompted the model to give a Social Phobia Inventory (SPIN) score based on the clinical interview, which was then compared to the ground truth value by means of a Pearson correlation.

#### Hypothesis

We hypothesized that Social Phobia Inventory (SPIN) scores inferred by GPT-4 after analyzing the interview transcript would show a high positive correlation (*r* = 0.5, according to the conventions from Cohen^40^) with the actual SPIN scores.

### Participants

Participants were recruited from the German general population via personal contacts of the experimenters, mailing lists, the course credit board of the Private University of Applied Sciences Göttingen, posters which were distributed on campus and in different locations around town, and social media platforms (Instagram, WhatsApp). Inclusion criteria were an age of 18 years and above, the ability to comprehend a questionnaire presented in the German language, and the capacity to provide informed consent and access to the necessary technical means to use the video conferencing platform. Exclusion criteria were non-compliance with the protocol, being under 18 years of age, inability to understand German, grave technical errors within the interview, and failure to complete the SPIN questionnaire. We ensured that participants were not paired with an interviewer within their social circle in order to guarantee anonymity. After completing the interview, participants were provided with their SPIN score if they wished to know it. All participants received general information about social anxiety, as well as resources for therapy, including phone numbers in case of emergency. Prior to the interviews, participants received written information about the purpose of the study, as well as the recording of the interview, and gave their consent according to the Declaration of Helsinki.

To test our hypothesis of a strong correlation (*r* = 0.5)^40^ with a statistical power of *1* − *β* = 0.95 and an ⍺-error probability of ⍺ = 0.05, a sample size of *n* = 38 participants was calculated as necessary, using the software G*Power^41^. Finally, a dataset consisting of *n* = 55 interviews was acquired. From this sample, *n* = 1 interviews had to be removed due to the participant revoking their consent. Another *n* = 3 participants did not fill in the SPIN questionnaire, which is why their interviews could not be considered for the study. This lowered the number of participants whose data could be evaluated to *N* = 51. Due to technical error, demographic data from *n* = 8 participants were missing; however, as their SPIN scores and interview data were complete, they were included in the calculations. Within the sample, *n* = 23 participants identified as male (45.1%, *M*_age_ = 32.83, *SD*_age_ = 13.71) and *n* = 20 identified as female (39.22%, *M*_age_ = 29.30, *SD*_age_ = 12.00). In general, participants’ ages ranged from 20 to 69 years old, with a mean score of *M* = 31.19 (*SD* = 12.91).

**Fig. 1:**
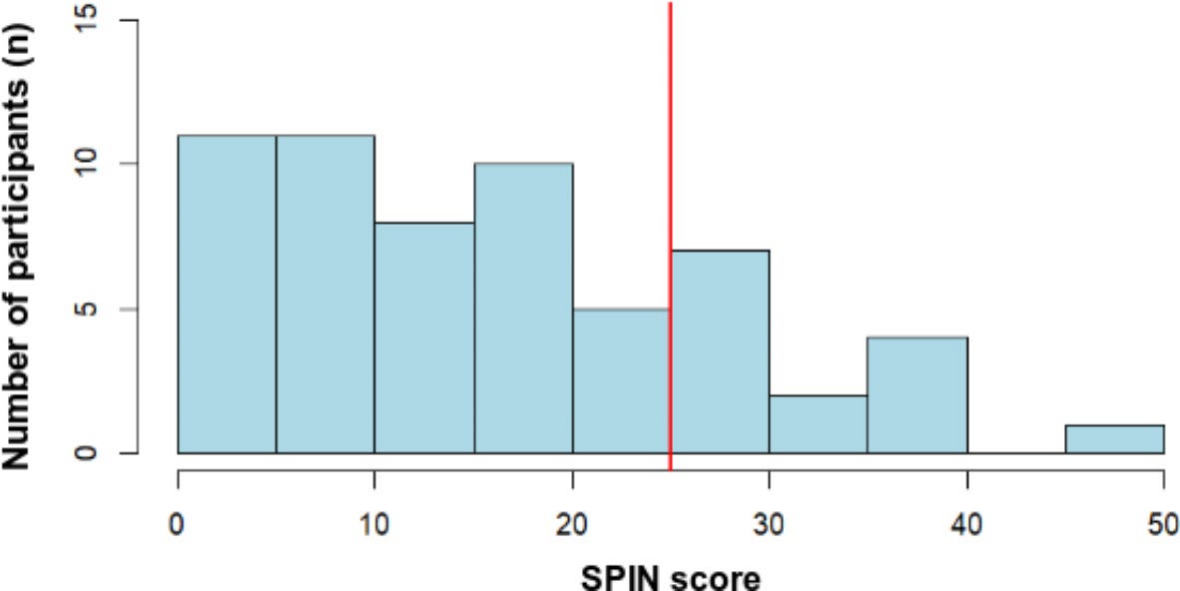
Frequency distribution of SPIN scores. Scores were obtained on the Social Phobia Inventory (SPIN) within the participant sample (*N* = 51). SPIN scores can range from 0 to 68. A cut-off score of 25 was determined to best differentiate between an unburdened and a psychiatric population in a German sample^43^.

Social anxiety symptom severity was measured as a participant’s self reported outcome with the German version of the Social Phobia Inventory (SPIN^42,43^; Stangier & Steffens, 2002). On average, the SPIN score was *M* = 17.08 (*SD* = 11.39), ranging from *min* = 0 to *max* = 50. *n* = 13 (25.4%) participants fell above the cut-off score determined by Sosic et al.^43^ for the German population, while the remaining *n* = 38 participants fell beneath this threshold. According to a Kolmogorov–Smirnov test, SPIN scores from the participants were distributed normally, *D*(50) = 0.12, *p* = 0.46. The frequency distribution of the participants’ SPIN scores is illustrated in Fig. 1.

### Ethics

Prior to participation, participants received written information on the purpose of the experiment, as well as of the interview and questionnaire process, the audio data collection, and data storage and protection. Informed consent for participation, as well as audio data collection and processing via AI, was obtained, in line with the Declaration of Helsinki, and saved in the audio recordings. The Ethics Committee of the PFH Private University of Applied Sciences Göttingen approved the research protocol under OS_18_200423/2 prior to the start of data collection. This study adhered strictly to GDPR regulations, ensuring that all data were handled fairly, ethically, and transparently while having adequate safety measures in place. Furthermore, we provided participants with the unconditional right to access, revise, remove, and limit the use of their personal data.

### Materials and instruments

#### Social Phobia Inventory (SPIN)

Participants filled in the German version of the Social Phobia Inventory (SPIN^42^) prior to their participation in the clinical interview. SPIN is a 17-item inventory that evaluates social anxiety symptom severity^44^. It refers to the past seven days, assessing fear, avoidance, and physiological symptoms. Answers are scored on a five point Likert-scale ranging from 0 to 4, where 0 indicates no symptoms and 4 indicates severe symptoms, leading to scores ranging from 0 to 68. In scale validation studies, psychometric properties were shown to be satisfactory, e.g., test–retest reliabilities between 0.78 and 0.89 were found, as well as Cronbach’s alpha ranges from ⍺ = 0.82 to ⍺ = 0.94. Moreover, in previous studies, an ideal cut-off score of 25 was found to achieve the optimal balance between sensitivity and specificity within the German population^43^.

#### Demographics

After filling in the SPIN, participants provided their demographic data (age, gender, education, German language literacy) in an online questionnaire. Due to technical error, demographic data (age, gender, German literacy, education) from *n* = 8 participants were missing.

#### Semi-structured social anxiety interview

The semi-structured interview was based on the Liebowitz Social Anxiety Scale (LSAS^45^), and contained a short stress test based on the Trier Social Stress Test (TSST^46^) towards the end. Since the TSST is not part of this research project, but will be used for further testing in another publication, we will not elaborate further. The LSAS is a questionnaire for evaluating the severity of social anxiety symptoms, usually administered and scored by clinicians^47^. It consists of 24 different situations featuring social interaction or performance, which are known to cause fear and avoidance in people with SAD. These situations are introduced and then rated on two 4-point Likert subscales for: (a) the level of anxiety experienced in them; and (b) the percentage of avoidance towards them. Psychometric properties for the original LSAS show a high internal consistency of Cronbach’s ⍺ = 0.95 for socially anxious patients and ⍺ = 0.92 for non-socially anxious people, and acceptable convergent and discriminant validity^47^. For this study, however, only 12 of the situations introduced by the LSAS were used to build an open-ended interview structure, which allowed us to collect qualitative data in the format of interview transcripts for GPT-4 to evaluate. Therefore, psychometric properties from the original version do not apply to our modified version. The rationale behind this modification was to: (a) allow for participants to share their experiences in social situations more freely, thus producing more content-related and linguistic cues, while (b) maintaining a structured format to ensure consistency and reduction of interviewer bias; and (c) to still be sufficiently short to keep the number of tokens manageable for the AI. The modification process was guided by a trained psychotherapist with expert knowledge in anxiety disorder research. Our modified, semi-structured social anxiety interview contained 12 of the original 24 situations from the LSAS. These 12 situations were selected based on: (a) the clinical features described in ICD-11^1^ for social anxiety disorder (SAD), including gender-related features (e.g. “urinating in public” for men); and (b) the factorial structure found by Caballo et al.^48^ . Scenarios referring to essential features from the ICD-11^1^ were included in our selection. Moreover, each factor identified by Caballo et al.^48^ was depicted in at least one item. To further encourage word production, interviewers described the scenario and then interviewed the participants about their typical behavior, thoughts, and emotions in this situation during the last week, using an open-ended question format. After this, participants were asked about the level of anxiety that they experienced in this situation and whether they had avoided it or would have preferred to avoid it. Interviewers received a script for the interview and were instructed to follow it in order to keep variance to a minimum. The average duration of the interview was 18 min (min: 8, max: 50).

#### GPT-4

GPT-4 is OpenAI’s most advanced model, with broad general knowledge and problem-solving capabilities^49,50^. It is a transformer-based architecture with the ability to generate text based on patterns learned from vast amounts of data during its training phase^50^. As the goal of our study was to test the model’s generalization capabilities in a zero-shot context, we did not fine-tune it for this specific task. In the initial stage of our approach, using API access, the model was prompted with a question of whether it was familiar with the Social Phobia Inventory (SPIN^42,43^) and how it is scored. The model responded affirmatively, indicating that data related to the SPIN^42,43^ have been used during its training phase. Due to OpenAI`s policy of not disclosing the specific datasets used during training, we were unable to verify this further.

### Process

Participants were recruited via social media, websites, personal contacts, posters placed on campus and throughout the town of Gottinga, mailing lists, and the course credit board of the PFH Private University of Applied Sciences Göttingen. Respondents sent an email to a contact person from the study team. The contact person provided each prospect with an invitation email that included a participant code for pseudonymisation, a link to an online version of SPIN^44^ , documents containing general information on the study, and consent forms for participation, data storage, and audio recordings. In the next step, participants could choose a time-slot from the interviewer’s schedule for their interview appointment. The interview took place on a secure video conference platform provided by the German Research Network. Participants were instructed to ensure anonymity by using their participant code instead of their name and leaving the camera turned off during the interview. Additionally, participants were instructed not to share any confidential information. The semi-structured interview for social anxiety described in section "Materials and instruments" was conducted and recorded. The interview was concluded with the TSST^46^. Interview records were stored safely on servers from the German Research Network, and could only be accessed and downloaded by a single experimenter from the research group at Reutlingen University. After downloading, interviews were automatically transcribed and translated from German to English, by running OpenAI’s Whisper Large-V2 model locally. Speaker annotation was done manually, as the state-of-the-art Pyannote’s Speaker Diarization did not show the capacity for this task. The part of the interview referring to the TSST was manually removed from the transcript. The GPT-4 model was prompted to infer the SPIN score for each participant, based on his/her interview transcript. A sample prompt is shown in Table 1. The interview transcript was formatted so that the interviewer‘s statements were prefaced with “Interviewer:” and the participant‘s responses were prefaced with “Participant:”. Due to the inherent variability in the outcomes of large language models (LLMs)^49^ and the temperature parameter being set to 0.3, which introduces variations in predicted scores, the average scores from three trials were considered.

**Table 1:** Example prompt.

Prompt	Analyze the following transcript from an interview with regards to symptoms of social anxiety disorder (SAD). Based on this interview, infer the score that this person has most likely attained on the Social Phobia Inventory, SPIN This score is in the range of 0 to 68, and if the score is greater than 25 then the person has social anxiety disorder. Only give me the score as an INTEGER WITHOUT EXPLANATION!
	[Interview]
	Interviewer: I have a test subject with the test subject number PFH238. Do you agree with the explanation of the agreement and with the recording of this interview? Participant: Yes.…
Output:	24

### Statistical analysis

To test our hypothesis of a high correlation between the actual SPIN scores and GPT-4 SPIN score predictions, a Pearson correlation was conducted, followed by a t-test for significance. Since demographic data and SPIN data were collected in two different surveys to ensure anonymity, the two datasets had to be fused by participant code. In two cases, two datasets existed for the same participant code. By comparing demographic data, it was determined that the codes were used by different participants; however, a comparison of timestamps could be used to correctly fuse datasets. Contact persons were asked to identify the correct interview by date. As only one interview was conducted, one of the double datasets could be removed, and the other participant could be correctly identified. The SPIN dataset included *n* = 80 participants, out of which* n* = 15 failed to fill in the whole questionnaire and were thus removed. Moreover: *n* = 3 datasets had to be removed due to invalid participant codes; *n* = 2 participant codes had to be removed, as they were double; in *n* = 4 cases, the interview did not take place; and in *n* = 1 case, the interview stopped abruptly after two minutes, which led to its removal. According to demographic data, no participant fell below the eligible age for participation or the minimum requirement of German literacy. Variables were tested for normality prior to testing correlations. Outliers were detected in the sample, but were not removed, since they are essential to test model capacity for determining severity in these cases.

### Metrics

We employed precision, recall, and F_1_ score metrics to evaluate the effectiveness of the classification model in this study. These are the most commonly used metrics for evaluating machine learning models with binary classifications and provide insights on the effectiveness of the model to make correct predictions^51^. Specifically, the percentage of true positives that the model accurately detects is termed *recall*, while the percentage of predicted positives that are actually true positives is called *precision*. The F_1_ score evaluates the overall performance of the model in classification tasks by harmonizing the scores of recall and precision. When interpreting the results of these metrics, higher scores mean better performance, and possible values are on the scale from 0 to 1. Further exploration of the results were done by using metrics of Pearson correlation coefficients (PCCs), mean square error (MSE), and coefficient of variation (CV). The degree and direction of a link between two variables were measured by the Pearson correlation coefficient. The coefficient has a range of − 1 to 1, where 0 denotes no correlation, and an absolute value close to 1 indicates a stronger relationship^52^. The average squared difference between the predicted and true scores is measured by the mean square error (MSE). A lower MSE indicates a more compact correlation between the predictions and the actual results, meaning more effective model performance. Lastly, the coefficient of variation is a statistical metric that is often used to evaluate the relative variability or dispersion of data. It is calculated as the ratio of a dataset‘s standard deviation to its mean, presented as a percentage^53^.

## Results

The statistical analysis was done in RStudio (Version 2024.04.02+764, R Version 4.4.0). Additionally, the packages psych and pROC were used.

### Descriptive data

In this dataset, GPT-4 predicted a score of *M* = 14.98 (*SD* = 12.73) across all participants. The scores ranged from min = 0 to max = 50. The prediction for male participants (*n* = 23) was *M* = 12.09 (*SD* = 11.52), while female participants (*n* = 20) had an average predicted score of *M* = 15.2 (*SD* = 11.34). Considering the cut-off of > 25, *n* = 12 (23.53%) participants were predicted to fulfill the criterion for social anxiety disorder (SAD). The remaining *n* = 39 (76.47%) participants were predicted to be below the threshold. The ground truth mean value (actual SPIN score) was 2.1 points higher (*M* = 17.08) than the value predicted by GPT-4 (*M* = 14.98), as depicted in Table 2. With a Cronbach’s α of 0.92, the SPIN scale performed as expected within our sample, showing an excellent internal consistency.

**Table 2:** Descriptive metrics for the comparison of the SPIN score and GPT-4’s prediction.

	M	SD	PCC	MSE	CV
GPT-4	14.98	12.73	0.792***	65.43	85.01%
Ground truth	17.08	11.39	0.792***	–	66.67%

Note: *** represents significance at *p* < .001.

### Prediction accuracy

With the t-test for the Pearson correlation of *r*(49) = 0.79 being significant (*p* < 0.001), we can reject the null hypothesis that there is no high correlation between participants‘ SPIN score and their SPIN score predicted by GPT-4. This means that the obtained correlation is most likely not due to chance. The value of the correlation itself (*r* = 0.79) can be interpreted as high according to Cohen’s conventions^40^. This means that the higher is the score that a person would receive on SPIN, the higher is the value that GPT-4 would attribute to that person.

**Fig. 2:**
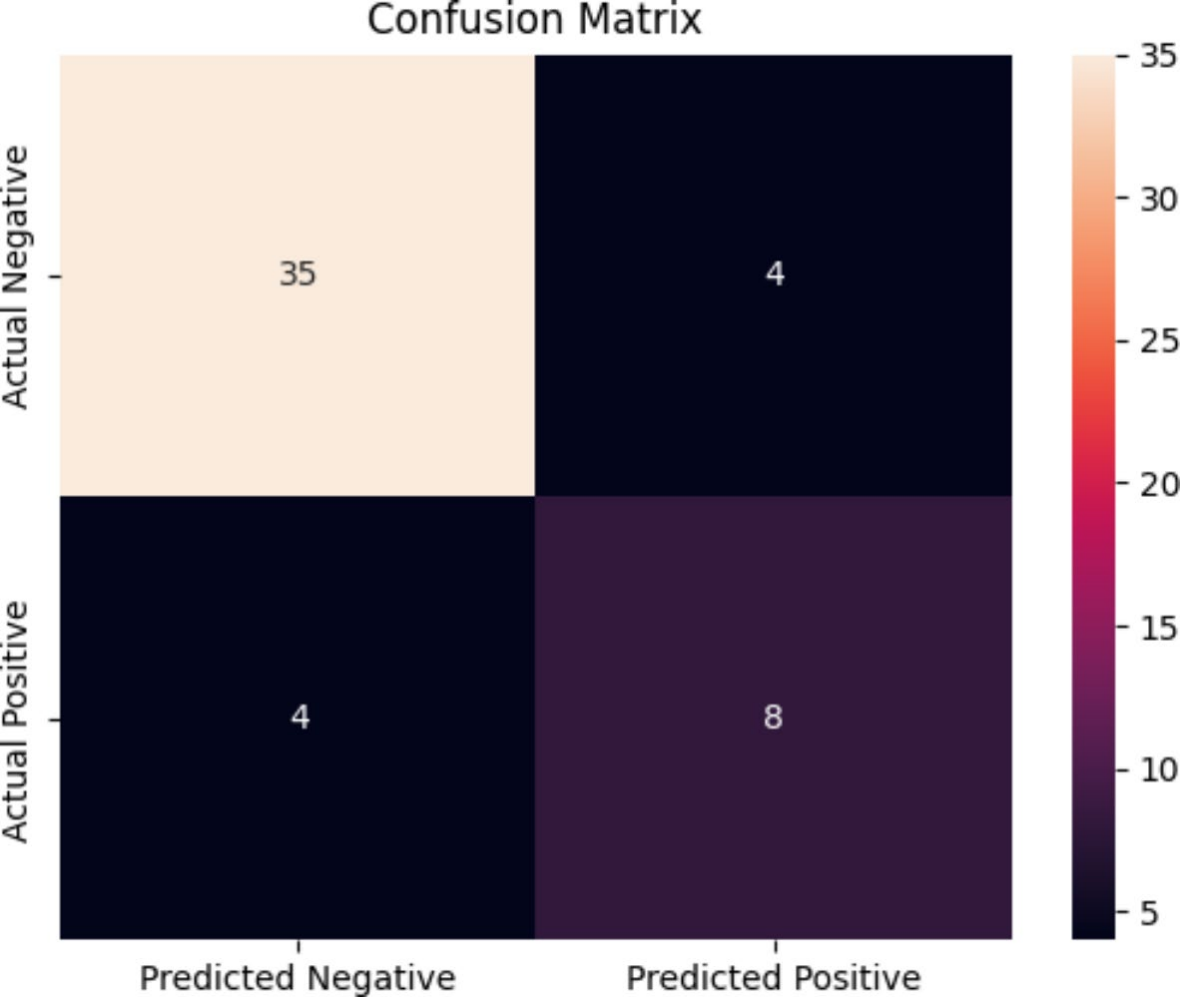
Confusion matrix showing *n* = size of each group. Ground truth refers to the actual SPIN score obtained by participants. Predicted refers to the score predicted by GPT-4.

As visually presented in Fig. 2, GPT-4 was able to categorize *n* = 35 true negatives and *n* = 8 true positives. Additionally there were *n* = 4 false positives and *n* = 4 false negatives. This corresponds to GPT-4 being able to predict the test scores with weighted precision, recall, and F_1_ score of 0.84, 0.84, and 0.84, respectively.

**Fig. 3:**
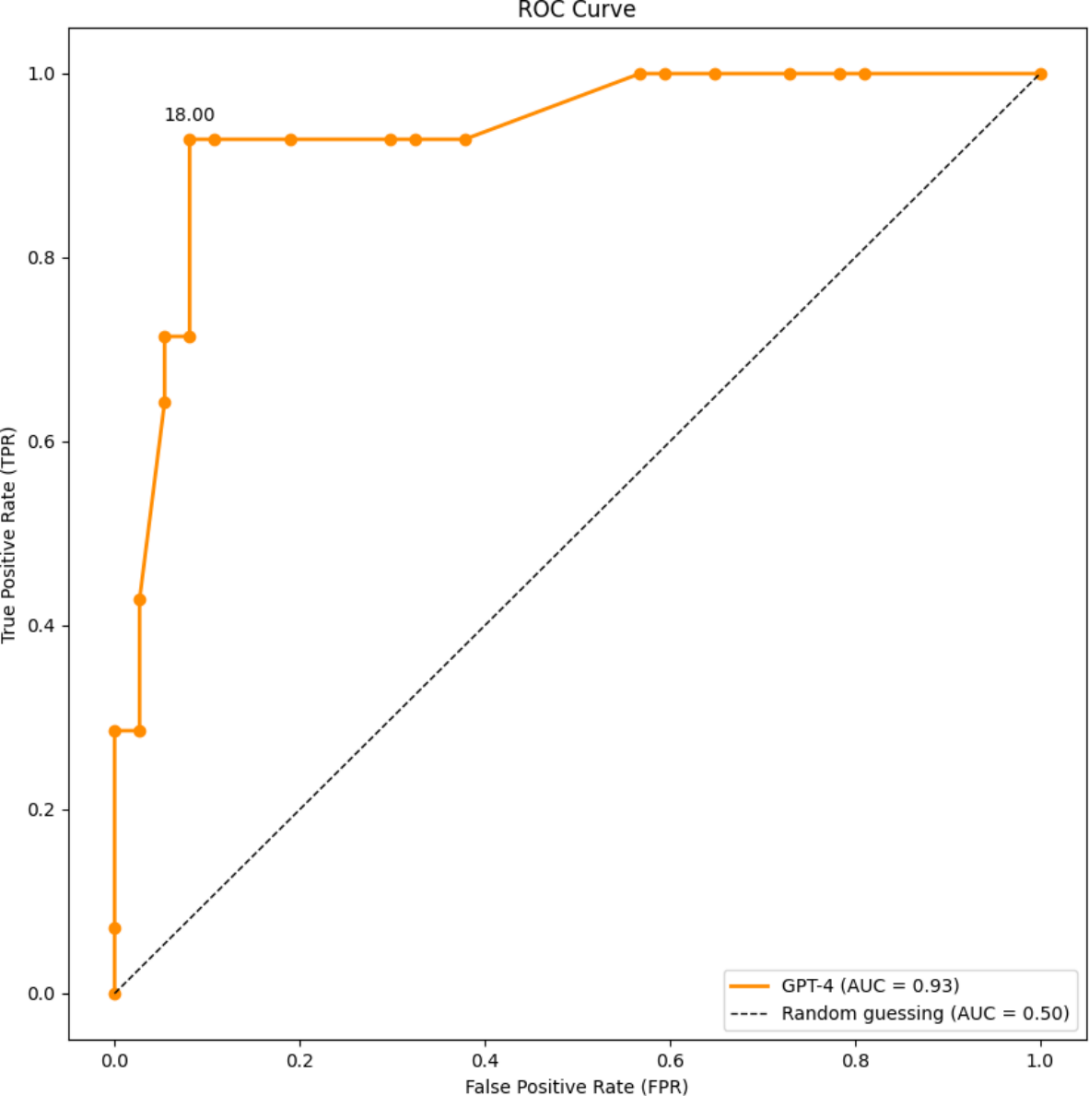
ROC curve showing the performance of the GPT-4 model.

The performance of the model was evaluated using the receiver operating characteristic (ROC) curve, as depicted in Fig. 3. The area under the curve (AUC) shows how accurate the classification is overall. A higher AUC reflects a better performance, with a value of 0.5 being as good as a categorization by chance. In this study, the AUC was 0.93, indicating excellent performance. This visualization of data enables an easier comparison of different screening thresholds based on the trade-off between the true positive rate (TPR) and the false positive rate (FPR). Based on this, a new optimal threshold of 18 was selected for classification of GPT-4 model predictions.

The classification metrics obtained by the GPT-4 model in predicting SPIN scores using the interview dataset are presented in Table 3. During the model evaluation process, two different thresholds were used as cut-off values to classify into positive and negative classes for the predicted scores. The threshold with a cut-off value of 25 was used based on previous work and recommendations from the literature based on a German sample^43^, while our ROC curve research revealed that the threshold of 18 was the most efficient. A potential explanation for such results is given in the discussion. Presented values include separate classification metrics in classifying positive and negative cases, as well as weighted values as aggregate measures including both classes.

**Table 3:** Classification metrics of GPT-4.

Classification metrics of GPT-4	Precision	Recall	F1
Threshold: 25			
Weighted	0.84	0.84	0.84
Positive cases	0.67	0.67	0.67
Negative cases	0.90	0.90	0.90
Threshold: 18			
Weighted	0.90	0.88	0.89
Positive cases	0.69	0.92	0.79
Negative cases	0.97	0.87	0.92

Note: Thresholds (25 and 18) represent cut-off values distinguishing positive and negative predicted classifications.

## Discussion

In this study, we investigated whether the LLM-model GPT-4 can detect and measure social anxiety in semi-structured clinical interview data, without any prior pretraining. For this, *N* = 51 semi-structured interviews (following the LSAS^45^) were conducted that described situations known to evoke social anxiety and avoidance in affected individuals, and asked participants to describe their behavior, cognitions, emotions, level of fear, and avoidance in this situation. The interviews were automatically transcribed and later evaluated by GPT-4, which was prompted to infer the participants’ score on the social anxiety self-report measure, SPIN. The score obtained from this prompt was later correlated with the participants’ actual SPIN score, as recorded in an online-survey prior to the interview.

We found a high correlation of *r(49)* = 0.79 between the score determined by GPT-4 and the actual SPIN-score. This is in line with our hypothesis. A high correlation between the inferred number and the ground truth is crucial for determining whether an LLM like GPT-4 has the potential to evaluate symptom severity. This is an essential first step for further research into the potential of AI systems for screening and diagnostic support systems.

Additionally, we tested whether GPT-4’s prediction of a conspicuous value held true in comparison with those inferred from the SPIN score. An F_1_ accuracy score of 0.84 was achieved, which is higher than the accuracy that Byers et al.^35^ obtained in an *n* = 10 sample of student veterans (61.2%), and falls within the accuracy range that Salekin et al.^34^ reported from stress test speech data in a large sample of students (68.3–90.1%). This underlines the potential of LLMs, such as GPT-4, for making inferences from speech data. However, it should be noted that GPT-4 showed higher recall and precision values for negative cases (recall for negative cases: 0.90) than for positive cases (recall for positive cases: 0.67) with the conventions for the sample population applied. This means that the system might not classify a participant high in social anxiety as conspicuous. Lowering the threshold to 18 strongly improved precision and recall. Further research on which factors contribute to a false negative attribution is warranted.

While social anxiety disorder (SAD) and depression constitute two different mental disorders which fall into different categories (anxiety disorders vs. affective disorders), it is still interesting to compare the results of this study with prior results that we obtained in a study on depression detection^25^. The accuracy score that GPT-4 managed to achieve for the detection of depression in *n* = 82 participants (*F*_*1*_ = 0.73) lies slightly lower than the one achieved for social anxiety within this study. Potential reasons for this might be: (a) the interview format applied in this study allowing for more text production and therefore more literary cues; (b) more content cues due to the interview format asking for behavioral, cognitive, and emotional cues; (c) a higher convergent validity between SPIN^41–43^ and our LSAS^45^-based interview than between HAMD and PHQ-9^54^ from the depression study^25^; (d) a higher amount of subthreshold participants in the dataset, which are easier for the system to identify; or (e) a combination of two or more of these factors. However, these are speculations, which should be further investigated in an explainable AI (XAI) approach.

### Strengths

Our study demonstrates the potential of GPT-4 for inferencing social anxiety symptoms from semi-structured interview data within the general population. One of the strengths of the study is the naturalistic distribution of the SPIN-scores around the cut-off value, meaning that it was particularly challenging for the system to discern cases. A value distribution like this is likely, however, when used as a screening tool. Using a semi-structured interview which allows for participants to freely elaborate on their experience with situations which are known to cause distress in people with social phobia came with the benefit of providing sufficient data for linguistic markers to be present while remaining close to the diagnostic criteria in content. Additionally, collecting our own dataset for a test procedure offers significant novelties for the research landscape. As an instance, it allows us to evaluate the GPT model‘s capacity for generalization while operating on an entirely unfamiliar set of data without prior fine-tuning. Furthermore, since no third-party or online platforms-originating dataset was used, it conforms with both ethical and legal guidelines on data handling and usage.

### Limitations

An important limitation of this study is that our ground truth value for social anxiety disorder (SAD) was derived from a self-report measure and not a professional diagnosis. This limits the validity of our findings, especially since no differential diagnosis has taken place. This might lead to our ground truth score being overly inclusive, e.g., towards individuals in which socially anxious symptoms can be attributed to another disorder (e.g., autism spectrum disorder, panic disorder, or generalized anxiety disorder) or does not fulfill the requisite time-criteria for a diagnosis of SAD. In addition, the inherent subjectivity of self-report measurements might have led to biased results, as the ground truth score, as well as the interview dataset, refer to the participant as a single source. Consequently, any measurements referring to “positive” and “negative” cases should be interpreted with caution, as they only refer to the cut-off criterion for SPIN. While SPIN has good psychometric properties (see section "Materials and instruments")^42,43^ and is highly accepted within research, as well as in clinical practice, scores above the cut-off criteria do not equal a diagnosis. Future studies are necessary to investigate whether the favorable results obtained within this study hold true if the ground truth is an expert rating (e.g., an actual diagnosis).

A further limitation of this study lies with the sample. Despite our participant group fulfilling the necessary sample size for hypothesis testing and including 25% of participants who scored above the cut-off score, we acknowledge that the range within the subgroup of burdened participants was limited: Only *n* = 4 participants achieved a SPIN score > 36, which was the mean SPIN score of the social anxiety group in the validation study by Sosic et al.^43^. While such values are not very common within the general population, more information from symptomatically burdened groups is needed to determine whether a measurement of symptom severity can be made in a clinical sample.

The sample is also relatively homogenous in terms of nationality, education and culture, as recruitment took place in a German university town and on campus. Therefore, it is possible that our results could potentially be inapplicable to people from other demographic backgrounds. It is important to verify our results for different samples, before generalized claims about the usefulness of NLP models in the detection of social anxiety symptoms can be made. Other limitations of our findings are that we: (a) did not evaluate cultural and ethnic background; and (b) had a high likelihood of overrepresentation of native German participants (only *n* = 2 participants did not identify German as their native language). As cultural differences exist in the presentation of social anxiety disorder (SAD)^2^, it is crucial to confirm results found within this study for different cultures. We used cut-off values that were obtained within a German sample^43^, which takes account of the limitation to this specific group; however, it is important to consider that the results regarding accuracy and correlation from this study might not apply to a different population. It is also relevant to note that model classification in our approach performs more effectively when the cut-off score is set at 18, achieving a weighted F1 score of 0.89, in contrast to 0.84 achieved with the cut-off score of 25 applied that was found to be ideal within a German population sample. This was discovered during the analysis of the ROC curve distribution scores. Such a disparity could be explained by an assumption that GPT-4 might be trained on SPIN datasets for the U.S. sample population, where studies found that the most efficient cut-off value is 19^42^. Further studies on specific population samples are encouraged to delve deeper into this disparity.

Another limitation in our approach is the limited range of demographic variables collected from participants. While essential information was obtained, such as age and gender, we did not account for other potentially influential factors, such as socioeconomic status, educational background, or ethnicity. This makes it difficult to interpret the external validity of our findings^55^. Future research should obtain a more diverse sample and evaluate the impact of culture, ethnicity, income, sexual orientation, and disability^55^ on model performance. Investigating whether AI-obtained results hold true in underrepresented groups is a prerequisite to prevent discrimination by these models in potential use cases. Therefore, the potential impact of such systems, particularly as it applies to vulnerable and underrepresented groups^56^, warrants its own investigation to prevent the replication of injustice with regard to the allocation of healthcare resources.

Concerning the informative value of the potential of NLP models, it is important to mention that only GPT-4 was tested; our past research showed vast differences between LLM models^25^. With regard to the relatively small dataset of *N* = 51, testing multiple models might have led to an inflation of alpha-error and therefore false positive outcomes, which is why we opted for testing a single hypothesis with a single model. The acquisition of a larger interview corpus is currently underway, granting us the participant numbers necessary to test the performance of different models, as well as fine-tuning in the near future.

As pointed out in our previous research^25,57^, paraverbal cues (e.g., intonation, speed, stuttering, etc.) and nonverbal cues (e.g., fidgeting, avoiding eye contact, etc.) were either not recorded (due to the camera being turned off) or not transcribed and could therefore not be considered in the evaluation. This constitutes a limitation of classical face-to-face clinical interviews. A multimodal approach with the capacity of including behavioral cues from multiple channels might be more effective. Meanwhile, our approach allowed for more anonymity, as the participants could not be seen, and this might have been less intimidating to people suffering from social anxiety than a face-to-face clinical interview.

While this study tested one of the mechanisms necessary for the creation of an automated interview system, the interviews were conducted by student interviewers. This limits our results with regards to an automated interview system potentially being a new factor. While it is essential to test different factors in the automation of clinical interviews one by one, it must be kept in mind that results from this study might not be applicable to a fully automated approach. As the interview is still conducted by a human interviewer, only the modality (online, with camera turned off) and the evaluative process are different. Whether an automated interview delivered by a chatbot or automated agent will lead to the same results is a topic for further research. In addition, whether such an automated interview will be perceived as less threatening by individuals experiencing social anxiety warrants further investigation. Another factor of considerable interest is whether the induction of social stress might lead to an improved detection of participants with social anxiety disorder (SAD). Results from Salekin et al.^34^, who used a speech stressor task to discern between students high and low in social anxiety, are promising in this regard. To test the potential of AI screening models for the detection of SAD from speech, we conducted a small stress test (TSST^46^) at the end of this study. We will continue to expand this dataset and evaluate whether: (a) online stress tests evaluated with AI lead to higher detection rates than evaluation from a clinical interview alone; and (b) whether evaluation by an AI system could replace the usual stress detection in the TSST, which relies strongly on biomarkers^46^.

### Future perspectives

Future research is necessary to validate the results obtained in this study by comparing model performance with diagnoses and/or symptom severity ratings by experts. The following should also be tested: (a) how the model performs in larger and more diverse datasets; (b) how different NLP models perform at this task; (c) whether pre-training can improve the diagnostic capabilities of the system; and (d) whether the interview can be conducted by a chatbot or automatic agent. Additionally, the issue of diminished help-seeking behavior in people with social anxiety disorder (SAD) and the potential for using online measures instead of fear-evoking face-to-face interactions^58^ should not just be considered from a diagnostic perspective. Research into interventions delivered via online-courses or apps already points towards favorable results^59^, and should be researched as a low-barrier starting point towards accessible mental health care.

### Practical implications

The results obtained within this study can be seen as a small, albeit promising, initial step towards evaluating the potential for NLP-based social anxiety detection. This warrants a deeper investigation with diagnoses obtained by expert raters. Given that model performance proves favorable in these scenarios, an AI-based SAD detection system could support clinicians in their diagnosis, helping to improve the current situation in which only 2.2% of cases presenting in clinics are diagnosed^16^ and only 23.6% received a diagnosis by a healthcare professional within 12 months^60^.

Studies with automated agents carrying out clinical interviews, such as Gratch et al.^61^, lend careful optimism to the potential of the interview being carried out without human intervention, and therefore potentially lowering the perceived stigma for people with social anxiety disorder to take part in a clinical interview.

## Conclusion

This study tested the capability of the LLM GPT-4 to infer social anxiety symptom severity from a semi-structured interview. A high correlation (*r* = 0.79) of the social anxiety predicted by GPT-4 with the actual self-report social anxiety score on the SPIN questionnaire proves the potential of this approach. However, our findings should be considered as a starting point for further research into the performance of different LLMs with different amounts of pre-training and the potential for an automated interview. A limitation lies with the ground truth in this study being a self-report measure and not a valid diagnosis of social anxiety disorder (SAD). An investigation comparing LLM results to actual clinical diagnoses constitutes a necessary next step.

## Data Availability

Data requests can be directed to the corresponding author via email. However, to protect participant rights, neither the transcripts nor the audio recordings will be released.
